# Alterations of monocarboxylate transporter densities during hypoxia in brain and breast tumour cells

**DOI:** 10.1007/s13402-012-0081-9

**Published:** 2012-06-15

**Authors:** Chang Cheng, Nina F. Jeppesen Edin, Knut H. Lauritzen, Ida Aspmodal, Stine Christoffersen, Liu Jian, Lene Juel Rasmussen, Erik O Pettersen, Gao Xiaoqun, Linda H. Bergersen

**Affiliations:** 1 Department of Anatomy/Pathology, Basic Medical College, Zhengzhou University, Science Road 100, Zhengzhou, Henan China; 2 Department of Brain and Muscle Energy Group, Institute of Basic Medical Sciences, University of Oslo, Oslo, Norway; 3 Department of Neuroscience and Pharmacology, University of Copenhagen, Copenhagen, Denmark; 4 Center for Healthy Aging, Faculty of Health Sciences, University of Copenhagen, Copenhagen, Denmark; 5 Department of Physics, University of Oslo, Oslo, Norway; 6 Department of Anatomy, CMBN, PO Box 1105 Blindern, 0317 Oslo, Norway

**Keywords:** Monocarboxylate transporter, Hypoxia, Glioblastoma, Breast cancer, Tumour cell

## Abstract

**Background:**

Tumour cells are characterized by aerobic glycolysis, which provides biomass for tumour proliferation and leads to extracellular acidification through efflux of lactate via monocarboxylate transporters (MCTs). Deficient and spasm-prone tumour vasculature causes variable hypoxia, which favours tumour cell survival and metastases. Brain metastases frequently occur in patients with advanced breast cancer.Effective treatment strategies are therefore needed against brain metastasis from breast carcinoma.

**Material and methods:**

In order to identify differences in the capacity for lactate exchange, human T-47D breast cancer cells and human glioblastoma T98G cells were grown under 4 % or 20 % oxygen conditions and examined for MCT1, MCT2 and MCT4 expression on plasma membranes by quantitative post embedding immunogold electron microscopy. Whereas previous studies on MCT expression in tumours have recorded mRNA and protein levels in cell extracts, we examined concentrations of the proteins in the microvillous plasma membrane protrusions specialized for transmembrane transport.

**Results:**

In normoxia, both tumour cell types highly expressed the low affinity transporter MCT4, which is thought to mainly mediate monocarboxylate efflux, while for high affinity transport the breast tumour cells preferentially expressed MCT1 and the brain tumour cells resembled brain neurons in expressing MCT2, rather than MCT1. The expressions of MCT1 and MCT4 were upregulated in hypoxic conditions in both breast and brain tumour cells. The expression of MCT2 also increased in hypoxic breast cancer cells, but decreased in hypoxic brain tumour cells. Quantitative immunoblots showed similar hypoxia induced changes in the protein levels.

**Conclusion:**

The differential expression and regulation of MCTs in the surface membranes of hypoxic and normoxic tumour cells of different types provide a foundation for innovation in tumour therapy through the selective targeting of MCTs. Selective inhibition of various MCTs could be an efficient way to quench an important energy source in both original breast tumour and metastatic cancer tissue in the brain.

## Introduction

Brain metastases are frequent in patients with advanced breast cancer, and effective strategies are crucial for treatment of brain metastases, particularly in this disease [1]. Characteristic of proliferating cancer cells is the low number of mitochondria in the cytoplasm and the high rate of cytosolic glycolysis, even in the presence of adequate oxygen. This so-called Warburg effect allows glucose moieties to be diverted into the synthesis of macromolecules needed for tumour cells proliferation [2–5]. Increased glycolytic rate leads to the accumulation of lactate and pyruvate in tumour cells, and elevated lactate levels correlate with a tendency to metastasize and a poor prognosis [6]. The levels of lactate and pyruvate in the cytoplasm need to be restricted, and are dependent on the regulation and expression of monocarboxylate transporters (MCTs) on the membranes of tumour cells [7, 8]. As a tumour grows, the distance to the blood vessels and thereby blood flow affect the local oxygen partial pressure and glucose concentration available to tumour cells. Sonveaux and collaborators have suggested that tumours contain oxygenated and hypoxic regions that interact through the consumption and production of lactate and that MCT1 inhibition have clinical anti-tumour potential [9]. In addition to MCT1, MCT4 and MCT2 also have the function of lactate shuttling [10]. We therefore hypothesize that the expression of MCTs may be different between hypoxic and normoxic tumour cells. Altered oxygen conditions in tumours can cause different mechanisms of metabolism. These metabolic modifications may be reflected by changes in MCT expression, and if sufficiently understood, could provide new targets for therapy. There have been no previous studies that have investigated the expression of different MCTs on the plasma membranes of hypoxic tumour cells compared with the expression in normoxic tumour cells. The aim of the present study was to determine whether cycling hypoxia (4 % O_2_ in the gas phase) leads to significant changes in the protein levels of different MCTs (MCT1, MCT2 and MCT4) on the plasma membranes of tumour cells. The protein densities were measured by quantitative post-embedding immunogold electron microscopy in two human cell lines, which were divided for a prevalent brain tumour (glioblastoma T98G) and a carcinoma (breast cancer, T-47D), respectively.

## Materials and methods

### Cell culture

Human T-47D breast cancer cells and human glioblastoma T98G cells (purchased from ATCC, LGC Standards AB, SE-501 17 Boras, Sweden) were grown as monolayer cultures in RPMI (Roswell Park Memorial Institute) 1640 medium (JRH Biosciences, Lenexa, KS, USA), supplemented with 10 % fetal calf serum (Gibco, Paisley, UK), 2 mM L-glutamine (SIGMA, St Louis, MO, USA), 200 units l^−1^ insulin (SIGMA), and 1 % penicillin/streptomycin (Gibco) at 37 °C in air containing 5 % CO_2_. The cells were cultured with two weekly reseedings, either in a normal incubator with about 20 % oxygen (ambient air with 5 % CO_2_), or in an IN VIVO_2_ 400 glove box hypoxia workstation (Ruskinn, UK) operated to contain 4 % O_2_ and 5 % CO_2_ in the gas phase. The pericellular oxygen concentration was measured using a Unisense Profix 3.0 programme (Unisense A/S, Aarhus, Denmark) for auto kinetic micro-sensor measurements, a motor controller and a motorized micromanipulator handling an OX10 Unisense micro-sensor (Clark-type) with a diameter of 25 μm, which was linked to a pico-ammeter as previously described [11].

### pH, lactate and glucose measurements

100 000 T98G and 500 000 T-47D cells were seeded and placed in an incubator or in the hypoxia glove box with 4 % O_2_. The pH was measured using a pH-meter from Sentron (Roden, the Netherlands). Lactate was measured on supernatant samples with a monitoring system (Powerlact, Med-TRONIK, Germany) with strips (Senslab, Germany) every day for 5 days. Glucose was measured on a monitoring system with strips (Anscensia Contour, Bayer Health Care, Germany).

### Preparation of cells for Western blotting and post-embedding immunogold electron microscopic analysis

The Western blotting for antibody testing was carried out on T98G and T-47D cells cultured in air containing 20 % oxygen. For post-embedding immunogold electron microscopy we used T98G and T-47D cells cultured in air containing either 4 % or 20 % oxygen. Cells were harvested by centrifugation at 15000 rpm for 10 min and stored in formaldehyde (4 %, freshly depolymerized from paraformaldehyde) and glutaraldehyde 0.1 %, freshly added in 0.1 M sodium phosphate buffer pH 7.4 at 4 °C until further processed.

### Primary antibodies

MCT1 M: Chicken anti MCT1 antibody was obtained from Millipore (USA). MCT2 G: Goat anti MCT2 antibody was obtained from GenWay Biotech (USA). MCT4 A: Rabbit anti MCT4 (SLC16A3) was obtained from Abcam (UK). MCT2 H and MCT4 H: Rabbit anti MCT2 and rabbit anti MCT4 were obtained from Professor A. P. Halestrap, University of Bristol, UK.

### Western blot analysis

Cells were harvested by trypsinization, rinsed twice with PBS and scraped into a RIPA buffer (Thermo Scientific Pierce, Germany) with a protease inhibitor (0.1 μM Aprotinin, 1.0 mM PMSF, 1 μM Leupeptin, 1 μM Pepstatin). Insoluble materials were removed by centrifugation and the protein concentration was measured. After boiling at 100 °C for 5 min in a SDS-loading buffer, equal amounts of protein per sample were separated by SDS-PAGE and transferred onto polyvinylidene difluoride transfer membranes (BIO-RAD, USA). The membranes were blocked with 5 % non-fat dry milk in PBST for 2 h at room temperature and reacted with the primary antibodies (diluted 0.5 μg/ml in PBST/5 % milk) at 4 °C overnight, followed by incubation with secondary HRP-conjugated antibodies. Immuno-complexes were visualized by enhanced chemiluminescence (GE Healthcare UK). The protein bands were quantitatively analyzed using Nikon image analysis software (NIS-Elements BR 3.1). All data concerning the level of specific proteins were normalized to the level of GAPDH. Western blotting experiments were repeated at least three times.

### Post-embedding immunogold electron microscopy

The procedure for post-embedding immunogold electron microscopy was adapted from Bergersen and colleagues [12]. The ultrathin sections were incubated overnight with primary antibodies against MCT1, MCT2 or MCT4 diluted in TBST containing 2 % HAS. The concentration of all MCTs were 5 μg/ml. Bound antibodies were visualized by incubating for 2 h with secondary (rabbit anti-chicken, goat anti-rabbit or rabbit anti-goat) immunoglobulin conjugated with 10 nm diameter colloidal gold (British Biocell International, UK). Sections were observed using a Tecnai 12 electron microscope. Pictures were taken randomly from the border region between two cells at primary magnifications of ×43 000.

### Quantitative immunogold analysis

**Fig. 1:**
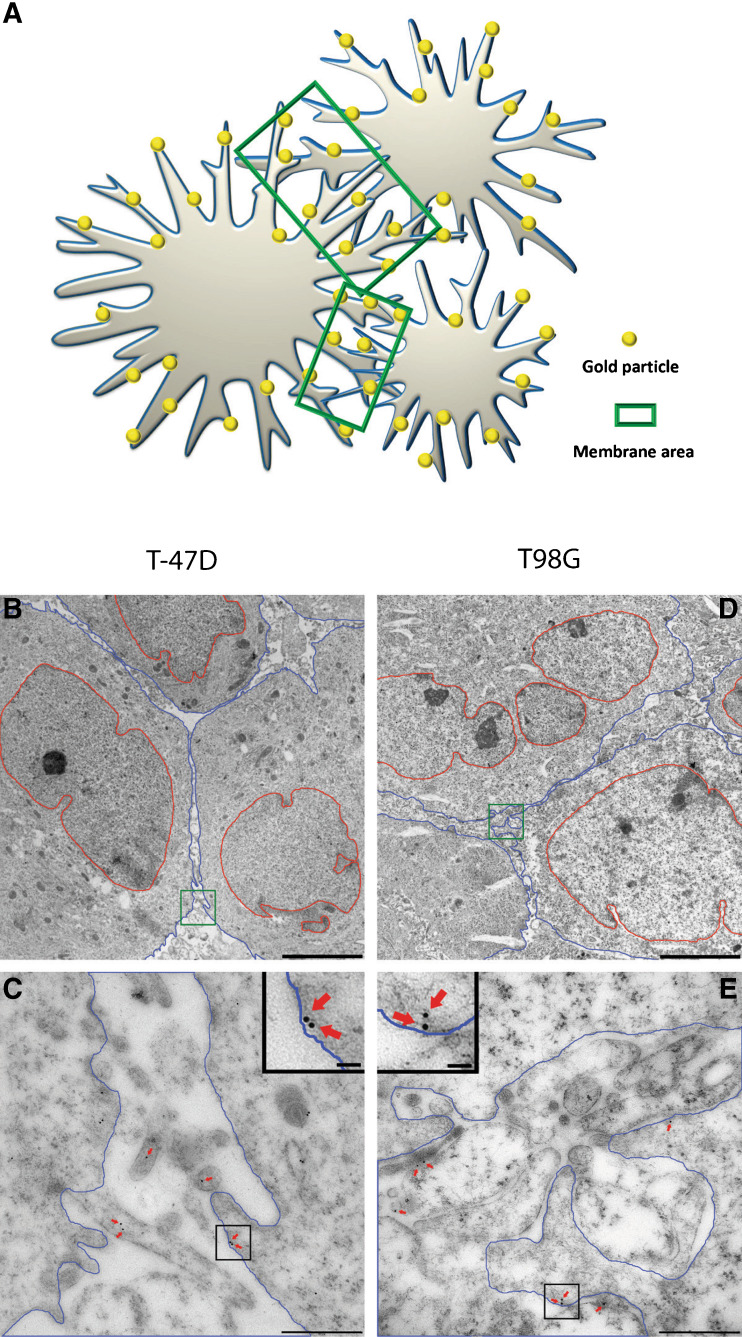
Electron micrographs of membrane area. **a** Illustration of the tumour cells (*grey*), gold particles (*yellow*) and membrane area (*green*). **b** Low magnification transmission electron micrograph from T-47D cells. **c** High magnification from (**b**) *green square*, cell membrane region. **d** Low magnification from T98G cells. **e** High magnification from (**d**) *green square*. The morphology is characteristic of tumour cells with nuclear atypia. The area between plasma membranes of two cells is covered with microvillous processes extended from the cell surface. *Blue area*, cell body; *Blue lines*, cell membranes; *Red lines*, nuclear membranes; *Green square*, membrane area; *Scale bars*, 5 μm in B and D, 500 nm in C and E; insets, 50 nm in C and E

A quantitative analysis of electron microscopic immunogold micrographs with clear plasma membranes were taken randomly from the sample. For each antibody solution (MCT1 M, MCT2 G, MCT2 H, MCT4 A and MCT4 H) and each of the four kinds of cells (T98G or T-47D cells grown with either 4 % or 20 % oxygen in the gas phase), 120 (MCT1 M, MCT2 G and MCT4 A) or 50 (MCT2 H and MCT4 H) micrographs were analysed. For each micrograph, cell membranes were marked by software ImageJ with Point density plug-in. The area between two cells creating a border region (Figs. 1 and 5, area between blue lines) was calculated and gold particles situated in the region, including 25 nm on each side of the membranes, were marked by the software [12]. This region contains most of the plasma membranes present in the samples, i.e. the transected plasma membrane covering the microvillous protrusions and the intervening areas of the tumour cell surface. The approximate density of transporters associated with the plasma membranes was calculated as the number of gold particles divided by the area.

In addition, 20 micrographs from each type of cell and antibody with a clear cytoplasmic area were taken out randomly, and the density of gold particles in this area without mitochondria was calculated, to compare them with the density at the plasma membrane.

### Statistical analysis

All quantitative data are represented as mean ± standard error of the mean, and the statistical significance was determined using unpaired two-tailed Student’s *t*-tests unless otherwise stated.

## Results

### Hypoxia

Cells cultivated under low-oxygen conditions were grown in a hypoxia box with 4 % oxygen in the atmosphere, which is close to the oxygen level in normal tissue (3–5 %) [13]. The cells need at least 2 weeks to adapt to the hypoxic conditions. At the time of the harvest and subsequent MCT analysis, the T98G cells had been grown with 4 % oxygen in the gas phase for 4 weeks and the T-47D cells for 2 weeks with two weekly reseedings. During this time the cells experienced cycling hypoxia as the pericellular oxygen level started at 4 % at each reseeding and ended below 0.1 % when the cell flasks were nearly full [14].

**Fig. 2:**
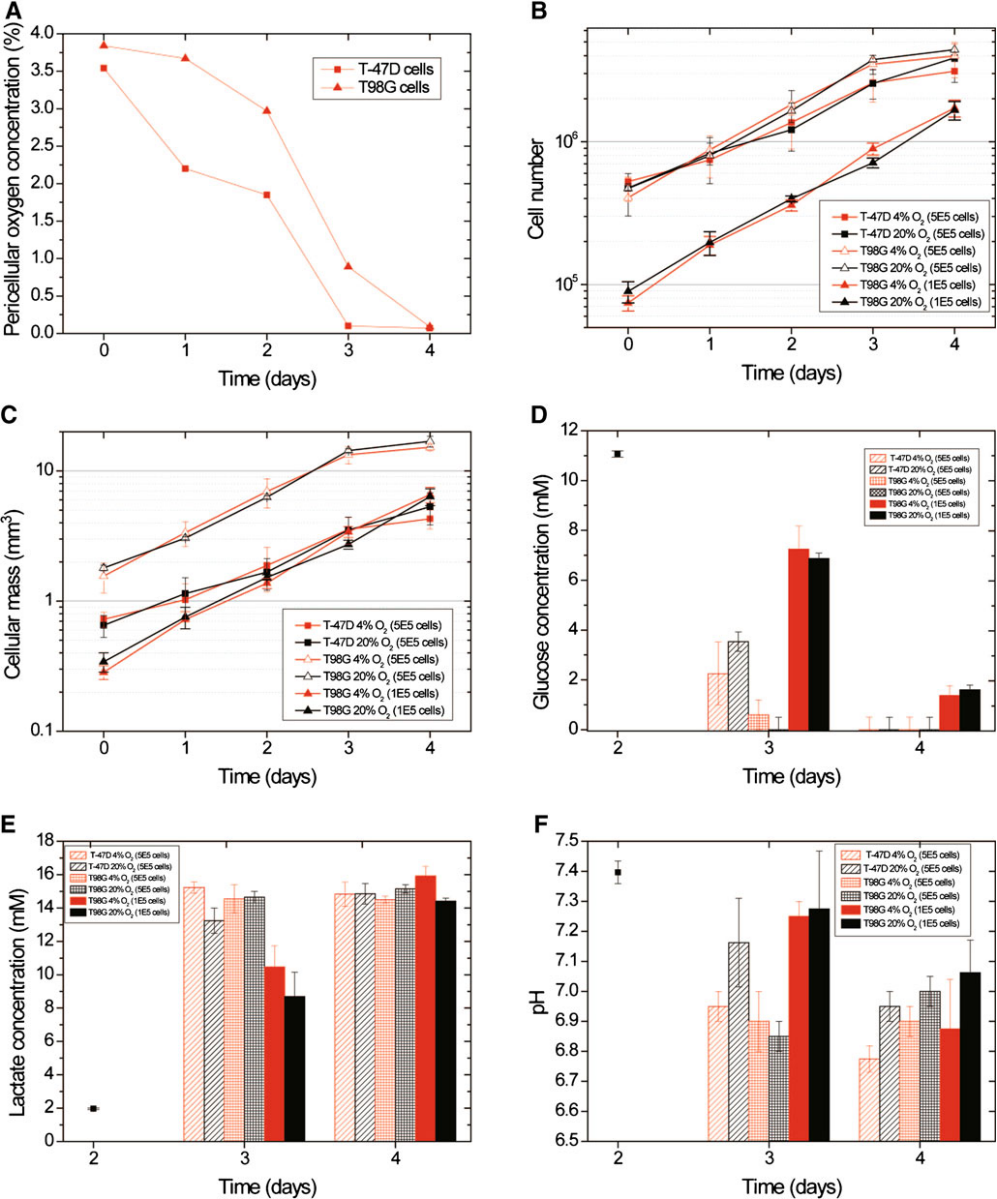
T-47D and T98G cells were grown either in 20 % or 4 % oxygen. The medium was changed on day 2. **a** The change in pericellular oxygen concentration as cell number increases between reseedings in cells flasks cultured with 4 % oxygen in the gas phase. Results from one representative experiment are shown. **b** T-47D cells and T98G cells were seeded at numbers resulting in full but not confluent flasks at day 4 for the MCT analysis. In addition measurements of lactate, glucose and pH were done in flasks with the same number of T98G cell as T-47D cells. **c** The cellular mass calculated from the cell volume and cell numbers from panel b. Panels **d**–**f** show the data after the medium change. **d** Glucose concentration (*black square* shows measurement of medium). **e** Lactate concentration (*black square* shows measurement of medium). **f** pH. *Error bars* show standard error of the mean from two parallel flasks

Figure 2 shows data from one such cycle and equivalent data for cells grown in ambient air. The change in the pericellular oxygen concentration as the cell number increased between reseedings is shown for cells cultured with 4 % oxygen in the gas phase (Fig. 2a). The T98G cells (median diameter 19.4 μm) are larger than the T-47D cells (median diameter 13.8 μm), and they also have a shorter doubling time (24 ± 2.4 h versus 28.8 ± 2.4 h). For that reason, the T98G cells were seeded at a lower number (∼10^5^) than the T-47D cells (∼5 x 10^5^), which is reflected in the longer time needed to reach the lowest pericellular oxygen concentration below 0.1 %.

The cells harvested for MTC analysis were grown in either 20 % or 4 % oxygen. The change in the total cellular mass in each flask (Fig. 2c) was calculated from the median cell diameter and cell number (Fig. 2b). The medium was changed on day 2 in order to provide enough glucose for further proliferation, and this influenced the measured lactate and glucose concentrations. As a result, the glucose, lactate and pH measurements are only shown after the medium change (Fig. 2d–f). The data include measurements of flasks seeded with the same number of T98G cells as used for T-47D cells (∼5 × 10^5^) as well as the number of T98G cells used in the MTC-analysis experiment (∼1 × 10^5^). The T-47D cells (when grown in both 4 % and 20 % oxygen) had used all available glucose on day 4 and the densely seeded T98G cells already exhausted the reserves about day 3. The sparsely seeded T98G cells did not consume all glucose (Fig.2d). The lactate production from day 2 to day 3 was higher in the flasks with the higher number of cells before all cell flasks reached approximately the same level on day 4. There was a tendency in both cell lines toward a larger lactate production in the hypoxic cells compared to normoxic cells on day 3 except in the densely seeded T98G flasks where the high level seen in all flasks on day 4 was already reached on day 3 (Fig. 2e).

The media of both cell lines reached a very low pH on day 4 in the flasks grown in 4 % oxygen (Fig. 2f). The densely seeded T98G cells and the hypoxic T-47D cells reached a low pH a day before sparsely seeded hypoxic T98G cells. There was a tendency towards higher pH in the cells grown in 20 % compared to 4 % oxygen except in the densely seeded T98G cells on day 3, but this was only statistically significant for T-47D cells on day 4 (*p* = 0.004).

### Specificity of antibodies and total protein levels of each individual MCT upon hypoxia compared with normoxia

**Fig. 3:**
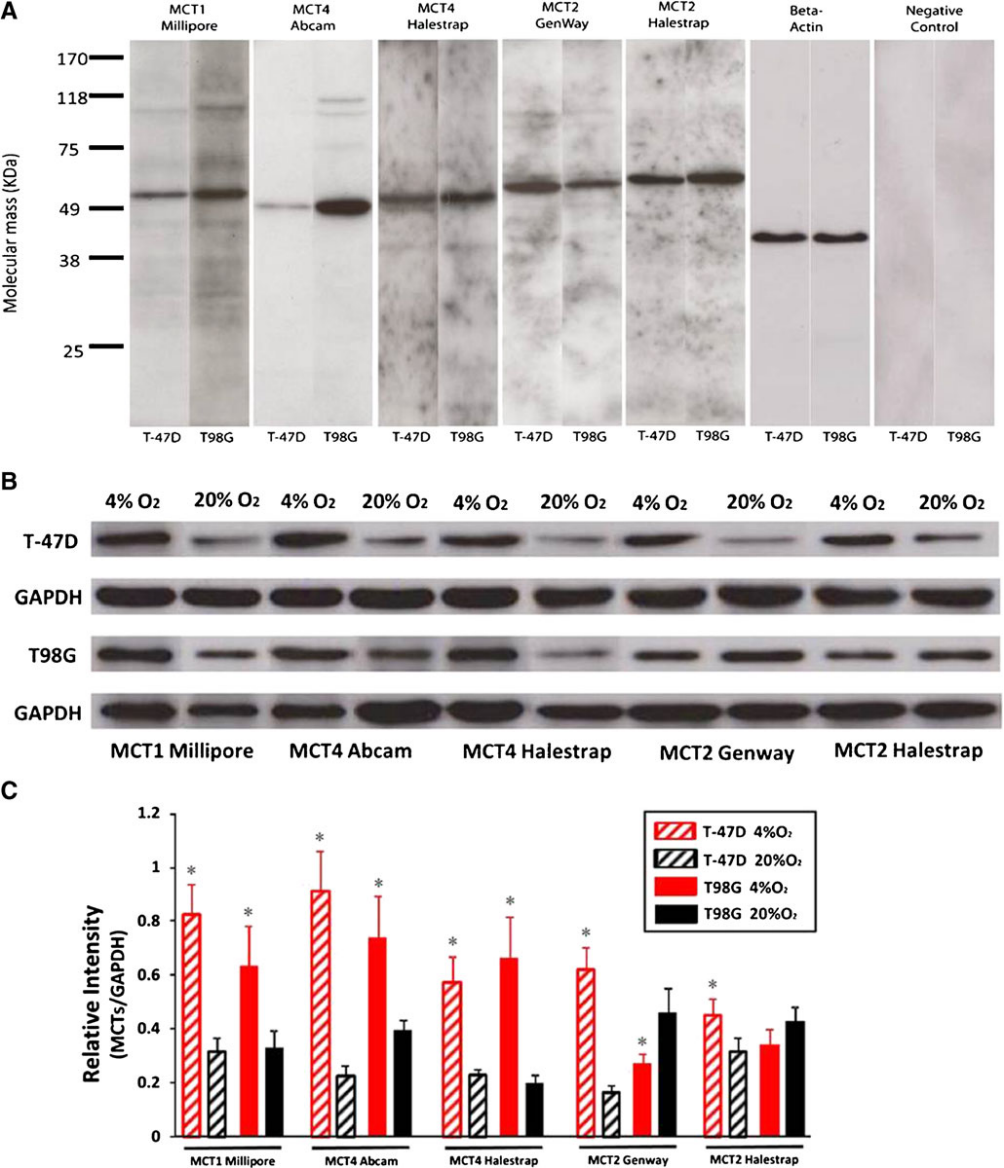
Antibody specificity and MCT protein quantification. **a** Immunoblots from T98G tumour cells and T-47D cancer cells gave for all antibodies single bands at a molecular weight of approximately 49–58 KD for MCT1 M (55 KD), MCT2 G (58 KD), MCT2 H (60 KD), MCT4 A (49 KD) and MCT4 H (52 KD). The results are consistent with the molecular mass of the proteins and the descriptions from the antibody suppliers. **b** The immunoreactive bands show the expression of each MCT in hypoxia and normoxia, in T-47D and T98G tumour cells. **c** The level of MCT1, MCT4 and MCT2 increased in T-47D cells after hypoxic cultivation, whereas a significant MCT2 expression decrease was detected in hypoxic T98G cells. *Error bars* show standard error of the mean. The *asterisk* indicates a statistically significant difference (*P* < 0.05) between hypoxia and normoxia

The antibodies to MCT1 Millipore, MCT4 Abcam, MCT4 Halestrap, MCT2 GenWay and MCT2 Halestrap showed single bands in immunoblots prepared from T98G and T-47D tumour cells. The positions of the bands are consistent with the reported molecular masses of the different subtypes of MCT transporters (Fig. 3a). Total relative MCT protein levels in cells were calculated by the ratio of MCT grey values to those for GAPDH loading control. MCT1 and MCT4 levels increased significantly in hypoxic T-47D and T98G tumour cells compared with normoxic cells, whereas MCT2 level increased in T-47D cells and decreased in T98G cells after hypoxic cultivation (Fig. 3b, c).

### MCTs distribution in normoxic or hypoxic T-47D and T98G cells

**Fig. 4:**
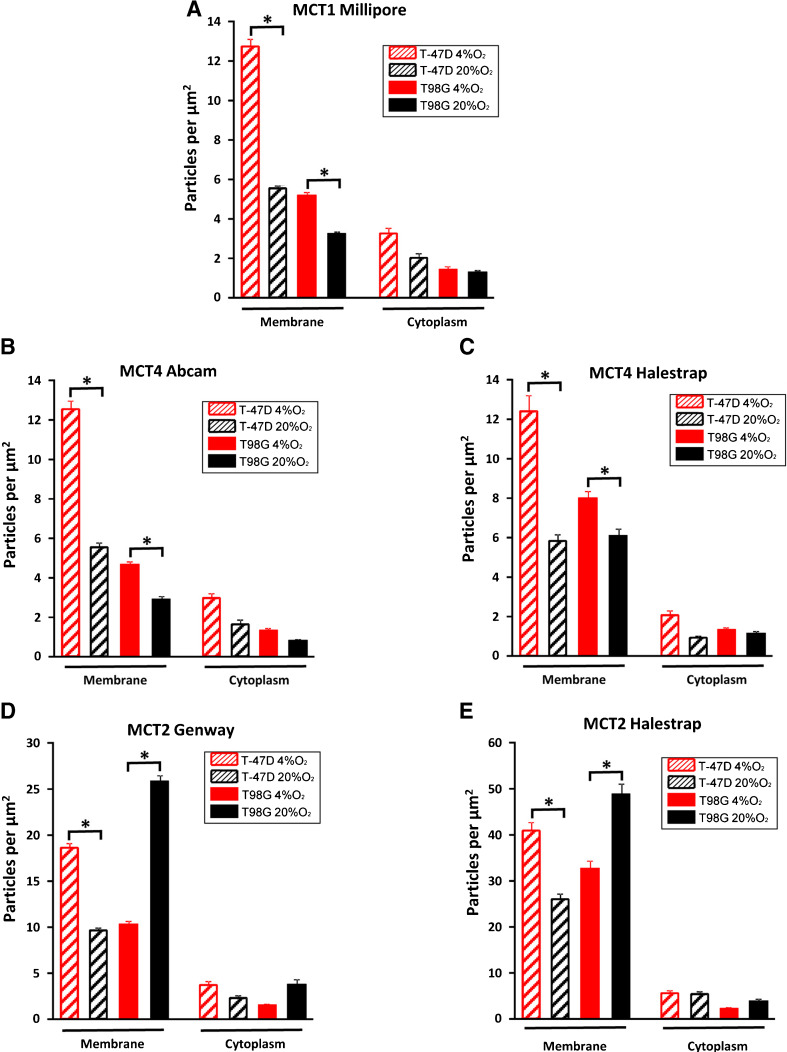
The density of MCTs on plasma membranes and cytoplasm. The density of MCTs on the membranes of hypoxic tumour cells and normoxic cells were significantly higher than that on cytoplasm areas without mitochondria areas. **a–c** The MCT1 and MCT4 density on membranes of hypoxic cells was significantly higher than that of normoxic cells in both T-47D and T98G cells. **d–e** The MCT2 density of T-47D hypoxic cells was significantly higher than that of normoxic cells. For T98G cells, it is the reverse. *Error bars* show standard error of the mean. *Asterisks* indicate statistically significant differences (*p* < 0.05) between hypoxia and normoxia

The presence of cellular MCTs in electron micrographs was displayed and quantified using immunogold analysis. For all of the MCTs, the densities were several times higher in the surface membrane regions compared to the cytoplasmic regions (Fig. 4a–e), despite the fact that the membrane area was diluted with extracellular space and intravillous cytoplasm. The MCTs were therefore highly concentrated in the plasma membrane.

**Fig. 5:**
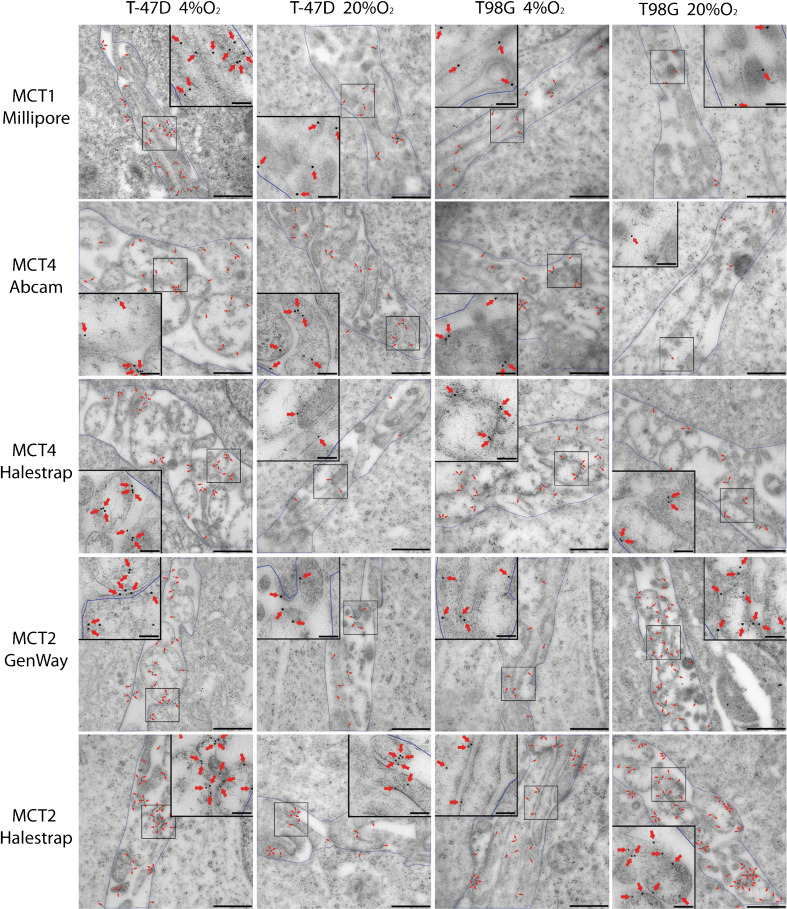
Electronic micrographs from T-47D and T98G cells with different MCTs. In electron micrographs, gold particles signalled immunoreactivity to MCT. To quantify the labelling of different transporter subtypes, the area between two cells creating a border region was calculated and gold particles (*red arrows*) situated in the region, including 25 nm on each side of the membranes (approximately the same distance as the lateral resolution of the immunogold method (Bergersen et al. 2008)), were marked by ImageJ software. The density of transporters’ immunoreactivity in the region containing microvillous processes of the surface membrane was calculated as the number of gold particles divided by the area. *Red arrows*, gold particles; Scale bars, 500 nm; insets, 100 nm

When normoxic and hypoxic cells were compared, the membrane density of MCT1 was significantly higher in cells grown with 4 % oxygen than in normoxic cells, both for T-47D and T98G cells (all *p* < 0.0001, Student’s *t*-test) (Fig. 4a and 5). The membrane density of MCT4 in hypoxic T-47D and T98G cells was significantly higher than that of normoxic tumour cells (all *p* < 0.0001, Student’s *t*-test) (Fig. 4b, c and 5). The antibodies from Abcam and Halestrap gave similar results.

In contrast, the density of MCT2 revealed a difference between T-47D and T98G cells. In T-47D cells, the density of MCT2 on plasma membranes in hypoxic cells was significantly higher than that of normoxic cells (all *p* < 0.0001, Student’s *t*-test). However, in T98G cells, the density of MCT2 on plasma membranes in cells grown with 4 % oxygen was significantly lower than that of normoxic cells (all *p* < 0.0001, Student’s *t*-test) (Fig. 4d, e and 5). Antibodies from GeneWay and Halestrap yielded similar results. The higher labelling intensity of the latter was associated with a lesser relative change between hypoxic and normoxic cells.

## Discussion

Through our studies, we have found that hypoxia causes changes in the densities of MCT proteins in the surface membrane protrusions of malignant tumour cells. In the breast cancer-derived cell line T-47D, all of the MCTs generally expressed in mammalian cells, i.e. MCT1, MCT2 and MCT4, showed upregulation under hypoxic conditions. During similar conditions, the glioblastoma-derived cell line T98G displayed MCT 1 and MCT4 upregulation, whereas MCT2 was downregulated.

These results suggest that tumour cells increase removal of lactate from glycolytic cells and increase lactate uptake by oxidative tumour cells. The question is what are the consequences of increased export and uptake of lactate? Increased export of lactate to medium lowers pH of the cellular environment and the extra-cellular matrix. This may influence remodelling of the matrix [15]. On the other hand, increased lactate uptake changes mitochondrial respiration. Increased mitochondrial respiration generates increased reactive oxygen species (ROS), increased oxidative damage to proteins and DNA, which generate genomic instability. Interestingly, increased ROS and mitochondrial dysfunction have been linked to malignancy in breast cancer [16, 17]. Decreased mitochondrial respiration lowers the ROS levels and generates genetic instability that is different from that previous described [18, 19]. This resembles induction of the Warburg effect [4] by ROS mediated inhibition of pyruvate kinase, which reduces pyruvate oxidation in mitochondria and thereby diverts glycolytic products into pathways promoting biosynthesis and increased ROS breakdown, both facilitating tumour growth [20].

This is the first study to demonstrate significant changes in the concentrations of MCT proteins in the plasma membrane of tumour cells. Specifically, we show that the changes occur in the microvillous region of the surface membrane, specialized for the exchange of solutes with the surroundings. Previous studies in various cell-types have recorded levels of protein in tumour tissues [21]. In the present study, we show that the concentrations of MCTs in cytoplasmatic areas are much lower than the areas containing plasma membranes, and also that they are less clearly different in normoxic and hypoxic specimens. Since the cytoplasmatic areas are large, the combined amount of MCTs in such an intracellular reservoir could be vast compared to the amounts inserted in or removed from the membrane in response to hypoxia, thereby explaining the lack of clear change observed in MCT densities in the cytoplasm. This may also explain the previously reported lack of hypoxia-induced changes in the expression of MCT proteins, when measured in cell extracts [22].

However, our immunoblots from extracts of cultured cells show that the hypoxia induced changes also comprise the total cellular levels of the MCTs, and therefore are not solely due to redistribution between membrane and intracellular stores. The low-affinity transporter MCT4 (Km about 35 mM) is generally associated with the export of monocarboxylates from cells, whereas the high-affinity transporters MCT1 and MCT2 are thought to primarily mediate the uptake from extracellular fluid. The observed hypoxia-induced upregulation of MCT4 in both cell lines is therefore in agreement with the demonstrated increased export of lactate to the medium. The upregulation of MCT1 in both cell lines, and of MCT2 in T-47D cells, may contribute to lactate efflux, but could also be viewed from the perspective of the proposed symbiotic relationship that exists within solid tumours between separate regions with different oxygen supply: normoxic cells close to blood vessels import and oxidize lactate released from hypoxic cells further away from vessels, thereby saving glucose the latter can use [9]. In addition, tumours typically undergo cycles of varying hypoxia [23], requiring cells to be prepared for episodes of net release of lactate alternating with episodes of net uptake to utilize the available oxygen for energy production.

The restricted oxygenation conditions in tumours bear some resemblance to those found in adipose tissue, particularly in the obese. Interestingly, hypoxia-induced changes similar to the ones observed by us in the glioblastoma-derived cells T98G were reported in adipocytes, i.e. the upregulation of MCT1 and MCT4 and the downregulation of MCT2 [15]. Adipocytes have recently been recognized to be akin to neuroendocrine cells, e.g. expressing the molecular machinery for neurotransmitter production [24]. As T98G cells are derived from glioblastoma tumours that originate in the nervous system and contain neural stem cells [25], they may be expected to behave more similarly to adipocytes than to the breast cancer-derived T-47D cells.

What can be the rationale for the downregulation of MCT2 in the T98G cells? A possibility is that the cells need to protect themselves from losing a critical substance or from the uptake of an adverse one. In this context, it is interesting that MCT2 has a higher affinity for pyruvate than the other MCTs, both in absolute terms and relative to lactate [26, 27]. The downregulation of MCT2 would therefore limit the loss of pyruvate to the medium, saving it for later conversion to lactate regenerating NAD^+^ from NADH or to metabolites supporting cell proliferation and growth. One additional possibility may be the necessity of regulating pH, which is central to the biology of tumours, notably for the ability to metastasize [28]. The high affinity of MCT2 for substrates may offer advantages over MCT1 and MCT4 in this respect.

It can be debated whether our choice of adjusting the cell numbers to result in a similar cellular mass was better than using similar cell numbers. On the one hand one can see that glucose is exhausted after 3 days in the densely seeded T98G culture, but not in the T-47D culture. This does not happen in the sparsely seeded T98G culture, thus to some extent supporting our choice. On the other hand the time course of pH in the densely seeded T98G cultures seems to be more in line with that of T-47D cultures than that of sparsely seeded T98G cultures. However, the cells were harvested after several cycles of hypoxia/reoxygenation and the treatment certainly resulted in different levels of the MCT-proteins in both cell lines compared to cell grown in ambient air. Interestingly, even if the lactate levels were the same in all cell flasks, the pH levels seemed to be lower in the hypoxic cell flasks (only statistically significant for T-47D cells). Plotting lactate as a function of pH (not shown) shows that the pH levels appear to continue to drop after a stabilization of lactate levels regardless of oxygen level. Thus, as the cells grow confluent, there must be other mechanisms responsible for acidification.

## Conclusions

The low affinity transporter MCT4, which is thought to mainly mediate the cellular export of monocarboxylates, was highly expressed by both the breast cancer-derived T-47D cells and the glioblastoma-derived T98G cells, and was upregulated in hypoxia in both cell types. The ubiquitously expressed high affinity transporter MCT1 was high in the breast cancer cells though low in glioblastoma cells in normoxia, but nonetheless was upregulated by hypoxia in both cell types. In contrast, the glioblastoma cells resembled neurons in expressing the high affinity transporter MCT2, rather than MCT1. Intriguingly, these cell down regulated MCT2 in hypoxia. Since MCT2 has a particularly high affinity for pyruvate, the observed down regulation may serve to prevent the leakage of pyruvate, conserving it for anaerobic energy production through conversion to lactate. The differential expression and regulation of MCTs in hypoxic and normoxic tumour cells opens possibilities for the innovation of tumour therapy through the selective targeting of monocarboxylate metabolism.
